# Correlation of bisphenol A and bisphenol S exposure with the metabolic parameters on FDG PET/CT image

**DOI:** 10.3389/fpubh.2024.1433122

**Published:** 2024-11-27

**Authors:** Liu Xiao, Xue Wen, Lin Li, Yuhao Li

**Affiliations:** ^1^Department of Nuclear Medicine, West China Hospital of Sichuan University, Chengdu, Sichuan, China; ^2^Department of Plastic and Burn Surgery, West China Hospital of Sichuan University, Chengdu, Sichuan, China

**Keywords:** BPA, BPS, FDG, liver SUV_max_, thyroid SUV_max_

## Abstract

**Purpose:**

Bisphenol A (BPA) and its analogs have been proved to be harmful to human health. This study aimed to assess the correlation of BPA and its major analog, Bisphenol S (BPS), with metabolic parameters within main organs using ^18^F-fluorodeoxyglucose positron emission tomography/computed tomography (^18^F-FDG PET/CT) imaging.

**Methods:**

A retrospective analysis was conducted on patients who had undergone FDG PET/CT imaging and were also examined for BPA and BPS levels. Urine samples were collected for detection of BPA and BPS. Standardized uptake values (SUV_max_ and SUV_mean_) of main organ tissues including liver, blood, spleen, muscle, thyroid, and cerebral cortex were quantified. Statistical analysis was performed using Spearman’s rank correlation.

**Results:**

Forty patients (20 female, 20 male; mean age: 56.1 ± 15.4 years) were included. Mean urine BPA and BPS concentrations were 2.1 ± 1.2 ng/mL and 1 ± 0.6 ng/mL, respectively. Urine BPA exhibited a moderate positive correlation with liver SUV_max_ (*r* = 0.351, *p* = 0.026) and SUV_mean_ (*r* = 0.361, *p* = 0.022) in male. No significant correlations were found between BPA and blood, muscle, spleen, thyroid, and cerebral cortex (*p* > 0.05). Conversely, urine BPS demonstrated a negative correlation with thyroid SUV_max_ in male (*r* = −0.43, *p* = 0.012) and SUV_mean_ (*r* = −0.432, *p* = 0.012), while a positive correlation was observed between BPS and cerebral cortex SUV_max_ in female (*r* = 0.366, *p* = 0.033).

**Conclusion:**

Urinary levels of BPA and BPS exerted distinct influences on tissue metabolic parameters observed via FDG PET/CT imaging, particularly affecting the liver, thyroid, and cerebral cortex.

## Introduction

Bisphenols (BPs) are synthetic chemicals utilized in the production of plastics, representing a group of widely distributed endocrine disruptors with potential environmental and human health hazards. Human exposure to BPs occurs regularly at chronic and low doses due to their ubiquitous presence in the environment, including food and water sources. Both BPA and BPS exhibit multi-system toxicity, encompassing reproductive, neurologic, hepatic, cardiovascular, and renal toxicities (1–3). Human bio-monitoring studies consistently demonstrate high detection rates of BPA in urine samples, reaching up to 90% in the general population (4, 5). Recent research has linked BPA and BPS exposure to elevated serum uric acid levels and an increased risk of hyperuricemia, with distinct dose–response relationships (6). At present, research mainly focus on effect of BPA and BPS on single tissue organ (7). These findings underscore the critical importance of timely and accurate monitoring of the effects of BPA and BPS on major organs.

^18^F-fluorodeoxyglucose positron emission tomography/computed tomography (^18^F-FDG PET/CT) imaging, a functional imaging modality, is widely employed in clinical practice for staging, re-staging, and evaluating treatment responses. Standardized uptake values (SUVs) serve as quantitative measures of glucose metabolism and are pivotal in interpreting FDG PET/CT images. Given the multi-system detrimental effects of BPA and BPS, we hypothesized that their impact on major organs could be visualized using ^18^F-FDG PET/CT imaging with SUV measurements. However, to date, no studies have analyzed the effects of BPA and BPS on SUVs in normal organs. Therefore, this study was conducted to assess the correlation of BPA and its major analog, BPS, with metabolic parameters within main organs utilizing ^18^F-FDG PET/CT imaging.

## Methods

### Patients population

A retrospective study enrolled patients who had undergone FDG PET/CT imaging and were subjected to BPA and BPS examination at our institution. BPA and BPS levels were assessed in urine samples. The collection of clinical samples/information in this study was approved by the ethics committee of West China Hospital of Sichuan University (No. 2023–1,095), and informed consent was obtained from all subjects according to the principles of the Declaration of Helsinki.

Standardized uptake value (SUV), such as maximum SUV (SUV_max_) and mean SUV (SUV_mean_) of normal main tissues, including the liver, blood, spleen, muscle, thyroid, renal and cerebral cortex, were measured. Patients with fever (*n* = 3), diabetes (*n* = 8), hematologic disorders (*n* = 4), abnormal liver or renal function (*n* = 5), primary or secondary hepatic, splenic, or aortic pathologies (such as neoplasms, large-sized cysts, aneurysms, inflammation, viral hepatitis B or C, and hepatic steatosis) (*n* = 11), were excluded from the study. Additionally, patients with FDG-avid tumors (*n* = 17) or who had received chemotherapy within 8 weeks prior to imaging (*n* = 1), radiotherapy targeting the liver or mediastinum (*n* = 5), or bone marrow colony-stimulating factor treatment within 2 weeks prior to imaging (*n* = 1), were also excluded. Finally, a total of 40 patients were included in the final analysis.

### Imaging technique

All subjects fasted for a minimum of 6 h prior to the examination to maintain low levels of glucose and insulin. ^18^F-FDG PET/CT images were acquired following the intravenous administration of 5.55 MBq (0.15 mCi) of ^18^F-FDG per kilogram of body weight via the cubital vein. PET scanning parameters were set at 4 mm/slice, while low-dose CT parameters were set at 120 kV, 40 mAs, and 5 mm/slice.

### SUVmax and SUVmean measurement in main organs

FDG uptake was measured by SUV, which were calculated according to the following the formula: (1) SUV_max_ = maximum activity in region of interest (ROI) (kBq/mL) / injected dose (MBq) × body weight (kg); (2) SUV_mean_ = mean activity in ROI (kBq/mL) / injected dose (MBq) × body weight (kg). Liver, blood, muscle, thyroid, renal and cerebral cortex were selected for analysis. The reason is SUV for these organs was easily measured and quantitatively evaluated. Quantitative analysis was conducted by placing a spherical volume of interest (VOI) with a diameter of 3 cm in the center of the right lobe of the liver, avoiding visible vessels on CT. Blood pool measurements were obtained by drawing a combined VOI on three contiguous slices within the thoracic aorta, measuring uptake within the vessel while excluding the vessel wall. Muscle measurements were performed by drawing a combined VOI on the quadriceps. Similarly, SUV_max_ and SUV_mean_ of the cerebral cortex (frontal lobe), thyroid, and spleen were measured using analogous methods.

### Statistical analyses

Statistical analyses were conducted using SPSS 22.0 (IBM SPSS Statistics, USA). Quantitative data were expressed as mean ± standard deviation (SD). Spearman’s rank correlation analysis was performed between each parameter and BPA and BPS levels. A significance level of *p* < 0.05 was considered statistically significant.

## Results

A total of 40 patients were included in the final analysis, comprising 20 females and 20 males, with a mean age of 56.1 ± 15.4 years. The mean concentrations of BPA and BPS were 2.1 ± 1.2 ng/mL and 1 ± 0.6 ng/mL, respectively. All the patients had normal blood glucose level with a mean concentrations of 5.7 ± 1 mmol/L.

All patients underwent ^18^F-FDG PET/CT scans to quantify SUV_max_ and SUV_mean_ in the main organs. Due to a history of thyroidectomy in 7 patients and the absence of head scans in 6 patients, thyroid SUV_max_ and SUV_mean_ were available for only 33 patients (SUVmax: 1.4 ± 0.37; SUVmean: 1.21 ± 0.28). Cerebral cortex measurements were obtained from 34 patients (SUV_max_: 11.04 ± 2.77; SUV_mean_: 9.68 ± 2.35). The liver demonstrated SUV_max_ and SUV_mean_ values of 2.71 ± 0.56 and 2.22 ± 0.44, respectively (Table 1). Blood glucose, height, weight and body mass index had no significant correlation with BPA and BPS (*p* > 0.05). All the patients were divided into two groups according to gender (group1: male group; group2: female group). Except for height and weight, there was no significant difference about age, BPA, BPS levels, SUV_max_ and SUV_mean_ in liver, blood, muscle, spleen, thyroid, cerebral cortex and renal (*p* > 0.05) (Table 1).

**Table 1:** Patients demographic characteristics and SUV data.

Variable	Value	Male group (*n* = 20)	Female group (*n* = 20)	*p*
Gender (number)	NA	20	20	NA
Age (year)	56.1 ± 15.4	56.0 ± 14.1	56.2 ± 16.9	0.968
Blood glucose (mmol/L)	5.7 ± 1	6.0 ± 1.1	5.6 ± 1.0	0.204
Height (cm)	161 ± 8	167 ± 5	154 ± 6	<0.001
Weight (Kg)	60.1 ± 12.9	67.6 ± 11.6	52.6 ± 9.6	<0.001
Body mass index (Kg/cm2)	23.05 ± 4.15	24.14 ± 4.37	21.95 ± 3.70	0.096
Urine BPA (ng/mL)	2.1 ± 1.2	2.4 ± 1.3	1.7 ± 1.0	0.068
Urine BPS (ng/mL)	1 ± 0.6	0.9 ± 0.6	1.0 ± 0.6	0.745
Liver SUV_max_ (g/mL)	2.71 ± 0.56	2.77 ± 0.64	2.64 ± 0.47	0.472
Liver SUV_mean_ (g/mL)	2.22 ± 0.44	2.23 ± 0.48	2.21 ± 0.42	0.830
Blood SUV_max_ (g/mL)	1.99 ± 0.43	1.99 ± 0.47	1.99 ± 0.38	0.98
Blood SUV_mean_ (g/mL)	1.67 ± 0.35	1.66 ± 0.39	1.67 ± 0.32	0.909
Muscle SUV_max_ (g/mL)	0.83 ± 0.17	0.88 ± 0.21	0.78 ± 0.11	0.069
Muscle SUV_mean_ (g/mL)	0.57 ± 0.088	0.58 ± 0.10	0.55 ± 0.07	0.283
Spleen SUV_max_ (g/mL)	2.19 ± 0.52	2.31 ± 0.62	2.07 ± 0.35	0.133
Spleen SUV_mean_ (g/mL)	1.79 ± 0.38	1.84 ± 0.45	1.75 ± 0.30	0.471
Thyroid SUV_max_ (g/mL)*	1.4 ± 0.37	1.47 ± 0.42	1.30 ± 0.27	0.195
Thyroid SUV_mean_ (g/mL)*	1.21 ± 0.28	1.24 ± 0.32	1.18 ± 0.20	0.569
Cerebral cortex SUV_max_ (g/mL)*	11.04 ± 2.77	10.29 ± 2.47	11.71 ± 2.91	0.138
Cerebral cortex SUV_mean_ (g/mL)*	9.68 ± 2.35	9.22 ± 2.09	10.08 ± 2.54	0.289
Renal SUV_mean_ (g/mL)	2.38 ± 0.39	2.31 ± 0.39	2.44 ± 0.39	0.266
Renal SUV_max_ (g/mL)	2.54 ± 0.38	2.49 ± 0.41	2.59 ± 0.39	0.440

SUV_max_, maximum standardized uptake value; SUV_mean_, mean standardized uptak value; NA, not available.

*thyroid SUV_max_ and SUV_mean_ were available for 33 patients; cerebral cortex SUV_max_ and SUV_mean_ were obtained from 34 patients.

**Figure 1:**
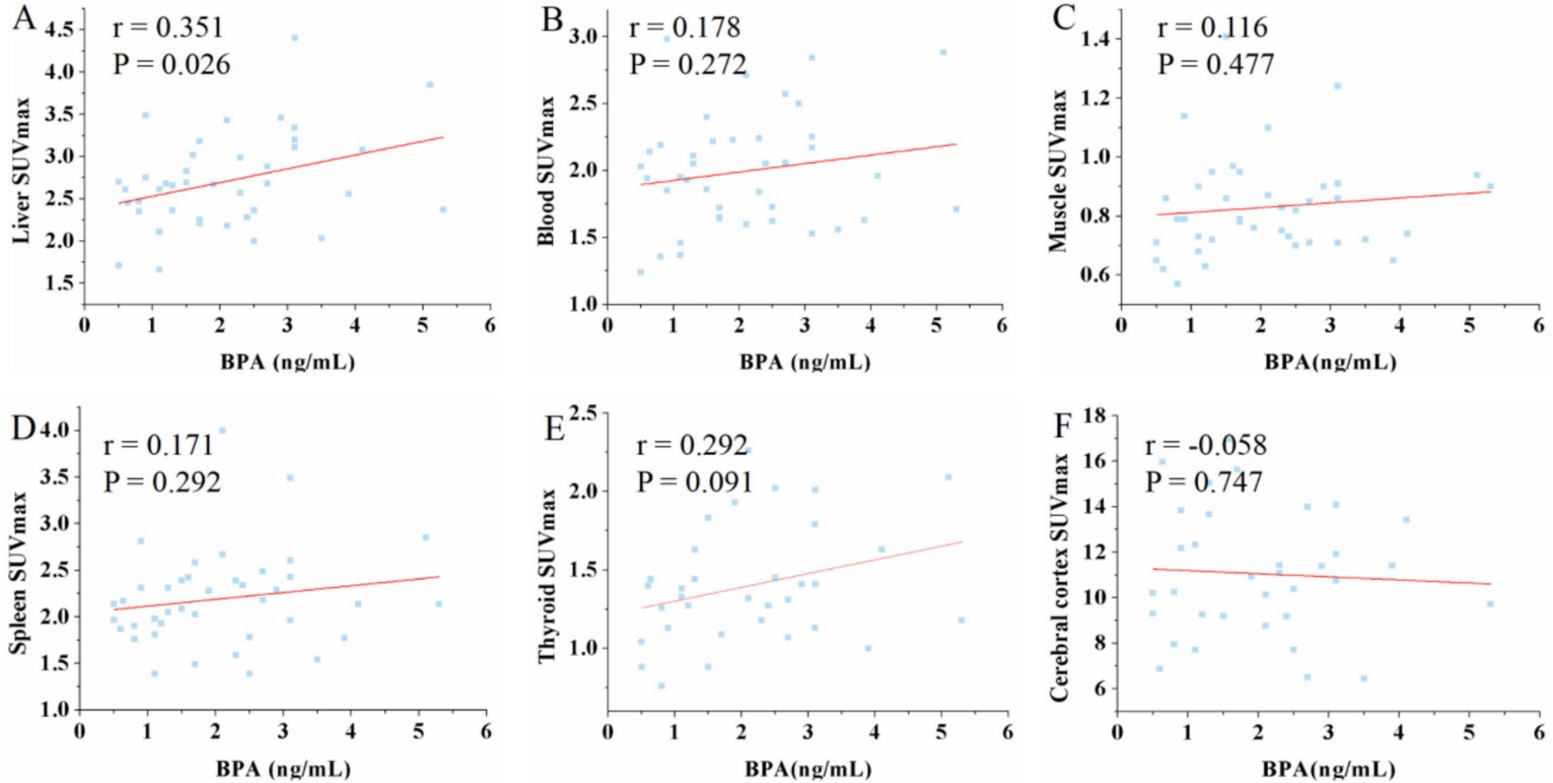
Scatter plots of the correlation between the BPA and maximum standardized uptake value (SUV_max_) in liver, blood, muscle, spleen, thyroid and cerebral cortex. **(A)** The urine BPA showed a moderate positive correlation with liver SUV_max_ (*r* = 0.351, *p* = 0.026). **(B–F)** The urine BPA showed no correlation with blood SUV_max_ (*r* = 0.178, *p* = 0.272), muscle SUV_max_ (*r* = 0.116, *p* = 0.477), spleen SUV_max_ (*r* = 0.171, *p* = 0.292), thyroid SUV_max_ (*r* = 0.292, *p* = 0.091) and cerebral cortex SUV_max_ (*r* = −0.058, *p* = 0.747).

**Figure 2:**
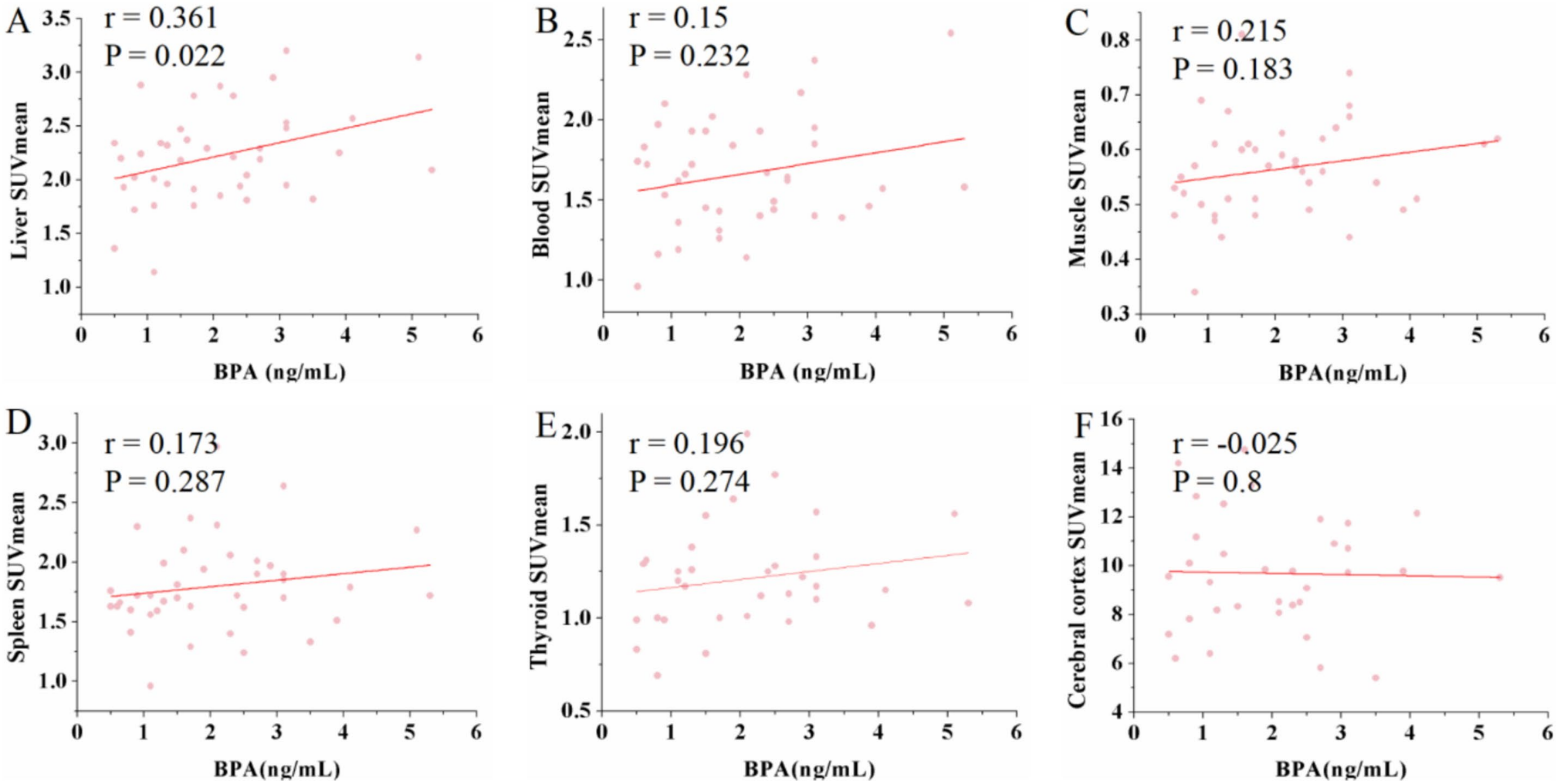
Scatter plots of the correlation between the BPA and mean standardized uptake value (SUV_mean_) in liver, blood, muscle, spleen, thyroid and cerebral cortex. **(A)** The urine BPA showed a moderate positive correlation with liver SUV_mean_ (*r* = 0.361, *p* = 0.022). **(B–F)** The urine BPA showed no correlation with blood SUV_mean_ (*r* = 0.15, *p* = 0.232), muscle SUV_mean_ (*r* = 0.215, *p* = 0.183), spleen SUV_mean_ (*r* = 0.173, *p* = 0.287), thyroid SUV_mean_ (*r* = 0.196, *p* = 0.274) and cerebral cortex SUV_mean_ (*r* = −0.025, *p* = 0.8).

The analysis revealed a moderate positive correlation between urine BPA levels and liver SUV_max_ (*r* = 0.351, *p* = 0.026) as well as SUV_mean_ (*r* = 0.361, *p* = 0.022). However, urine BPA levels had no significant correlation with renal SUV_max_ (*r* = −0.055, *p* = 0.735) and SUV_mean_ (*r* = −0.061, *p* = 0.707). No significant correlations were observed between urine BPA and SUV_max_ or SUV_mean_ in blood, muscle, spleen, thyroid, and cerebral cortex (*p* > 0.05) (see Figures 1, 2).

**Figure 3:**
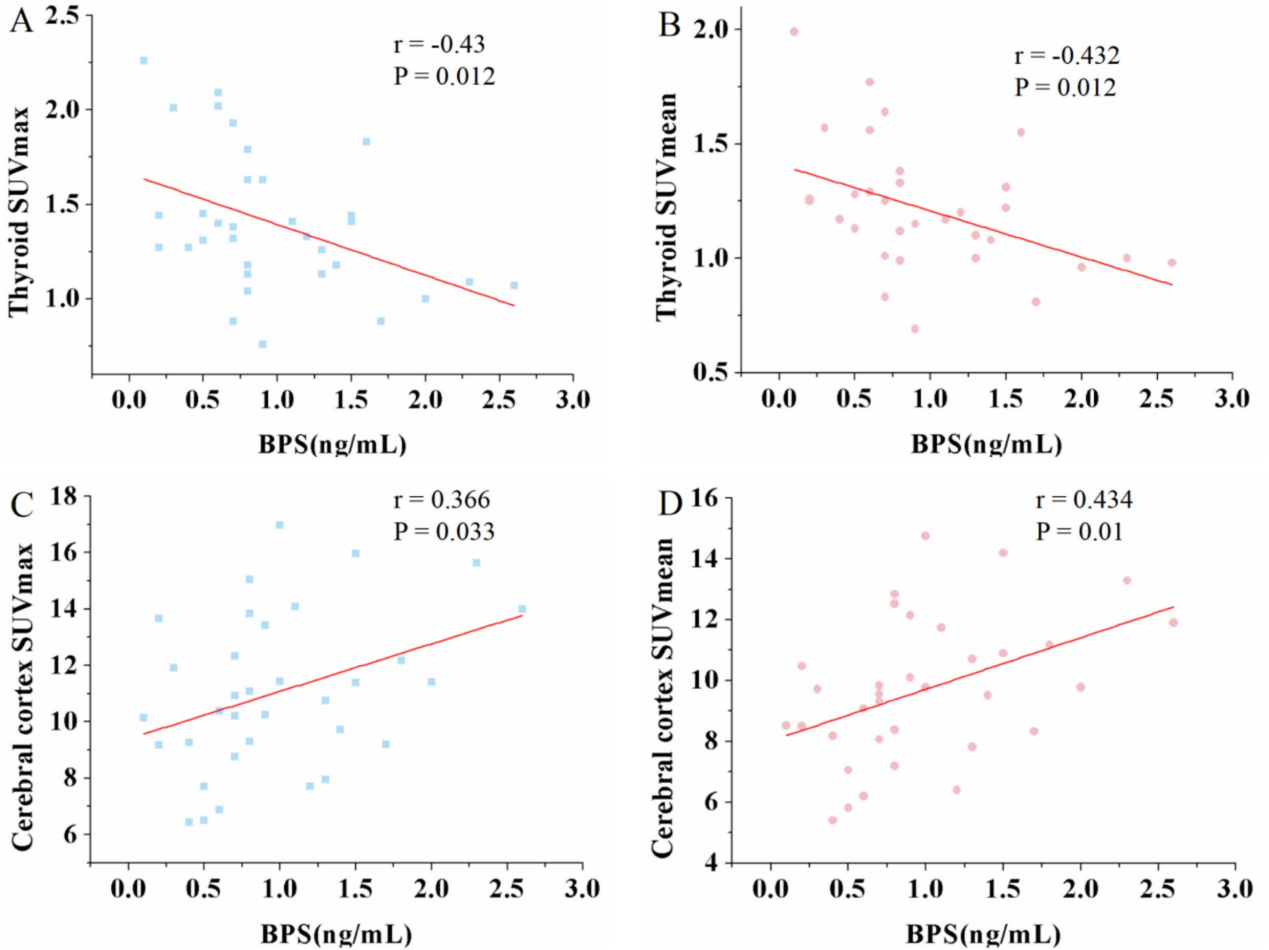
Scatter plots of the correlation between the BPS and maximum standardized uptake value (SUV_max_), mean standardized uptake value (SUV_mean_) in thyroid and cerebral cortex. **(A,B)** The urine BPS showed a moderate negative correlation with thyroid SUV_max_ (*r* = −0.43, *p* = 0.012) and SUV_mean_ (*r* = −0.432, p = 0.012). **(C,D)** The urine BPS showed a moderate positive correlation with cerebral SUV_max_ (*r* = 0.366, *p* = 0.033) and SUV_mean_ (*r* = 0.434, p = 0.01).

Conversely, urine BPS exhibited a negative correlation with both thyroid SUV_max_ (*r* = −0.43, *p* = 0.012) and SUV_mean_ (*r* = −0.432, *p* = 0.012). Additionally, a positive correlation was observed between urine BPS and cerebral cortex SUV_max_ (*r* = 0.366, *p* = 0.033) and SUV_mean_ (*r* = 0.434, *p* = 0.01) (see Figure 3). Similarly, no significant correlations were found between urine BPS levels and SUV_max_ or SUV_mean_ in the liver, blood, muscle, spleen and renal (data not shown).

**Figure 4:**
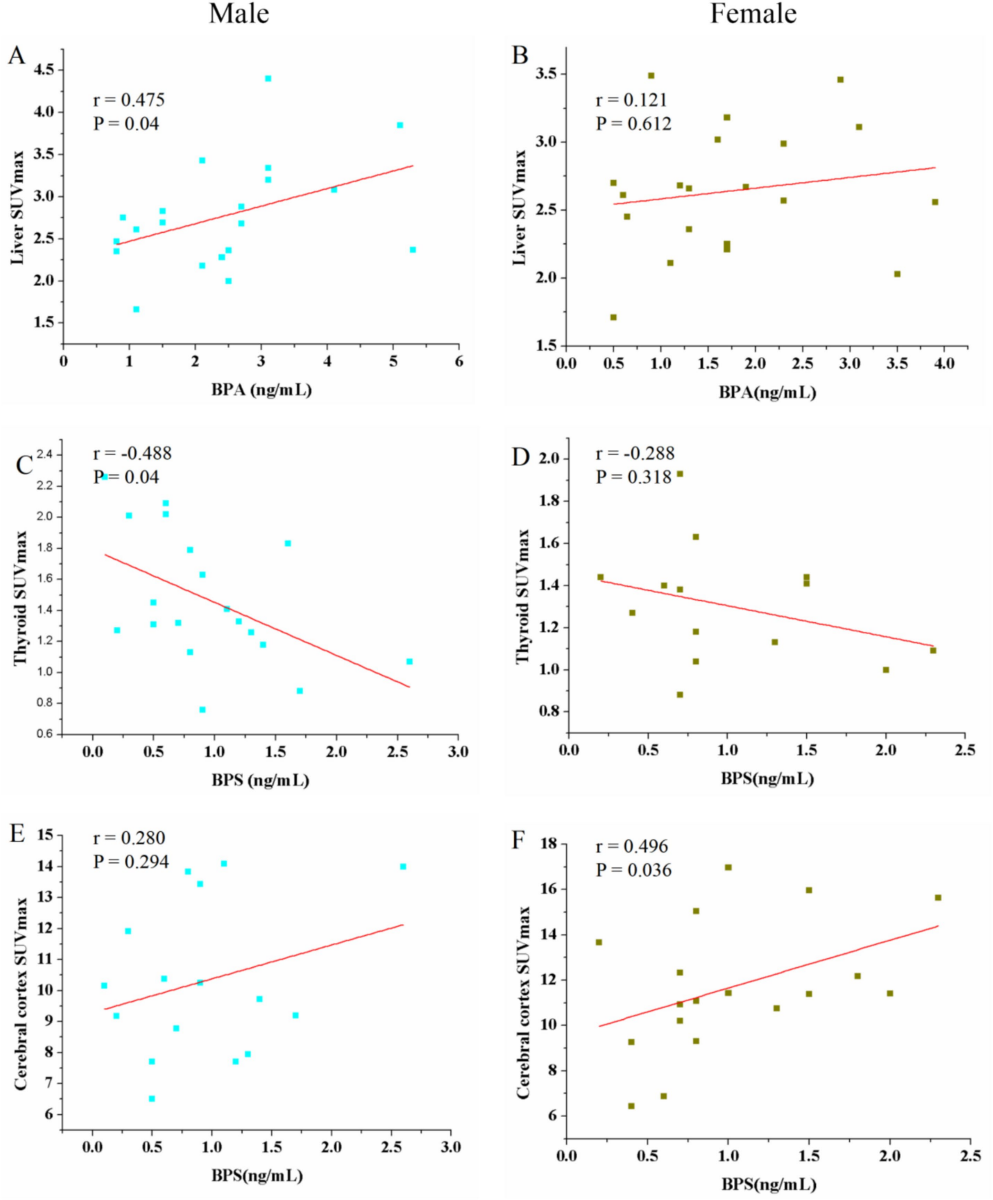
Scatter plots of the correlation between the BPA, BPS and maximum standardized uptake value (SUV_max_) in liver, thyroid and cerebral cortex in male and female patients. **(A)** The urine BPA showed a positive correlation with liver SUV_max_ in male patients (*r* = 0.475, *p* = 0.04). **(B)** Female had no significant positive correlation (*r* = 0.121, *p* = 0.612). **(C–F)** The urine BPS showed a negative correlation with thyroid SUV_max_ in male patients and a positive correlation with cerebral cortex SUV_max_ in female patients; no correlation between BPS and thyroid SUV_max_ in female, and between BPS and cerebral cortex SUV_max_.

It is reported that BPA and BPS had sex-dependent disruptive effect (8). Thus, correlation between BPA, BPS and SUV_max_ in different tissue organs in male and female patients was analyzed. The urine BPA showed a positive correlation with liver SUV_max_ in male patients (*r* = 0.475, *p* = 0.04) (Figure 4A). However, female had no significant positive correlation (*r* = 0.121, *p* = 0.612) (Figure 4B). However, no significant correlations were observed between urine BPA and SUV_max_ in blood, muscle, spleen, thyroid, cerebral cortex and renal (*p* > 0.05). The urine BPS only found a negative correlation with thyroid SUV_max_ in male patients (*r* = −0.488, *p* = 0.04) and a positive correlation with cerebral SUV_max_ in female patients (*r* = 0.496, *p* = 0.036) (Figures 4C–F).

## Discussion

To our knowledge, this study represents one of the initial investigations into the relationship between BPA, BPS, and SUV_max_ and SUV_mean_ in main organs as observed on FDG PET/CT imaging. Our findings revealed BPA and BPS had sex-dependent effect for different organ tissue, which manifested a positive association between urine BPA concentration and liver SUV_max_ and SUV_mean_ in male, while BPS demonstrated a negative correlation with SUV_max_ and SUV_mean_ in the thyroid in male and a positive correlation in the cerebral cortex in female.

SUV, as a functional index, is a very important quality index of FDG PET/CT studies at the PET/CT image interpretation stage, which reflects glucose metabolism. In clinical practice, a five-point scale (5-PS) model can be used with liver and blood pool SUV measurements to differentiate abnormal FDG uptake from physiological FDG uptake. These measurements are often used as reference background values to distinguish tumors and define treatment response. Thus, it is imperative to analyze possible influencing factors for SUV value. From perspective of public health, a comprehensive analysis of the relationship between BPA and BPS and SUV is helpful to quantitatively evaluate the effects of BPA and BPS on important organs. SUV derived from FDG uptake by these organs on ^18^F-FDG PET/CT imaging may reflect the extent of BPA and BPS exposure in the body.

It is reported that BPA and BPS had sex-dependent disruptive effect (8). This may be attributed to the difference in bisphenols metabolism, estrogen receptor expression, as well as gender-related bisphenols exposure. Gender-related hormonal variations may lead to different responses to bisphenols exposure (9). Our study suggests that increased BPA exposure in male may lead to heightened FDG uptake in the liver. It is known that BPA undergoes hepatic metabolism, primarily as glucuronidated metabolites, and is subsequently excreted through urine (10). Additionally, research by Jia et al. utilizing non-targeted metabolomics has indicated potential effects of bisphenol analogs on mitochondrial function attenuation and enhanced glycolysis, providing a theoretical framework to elucidate the observed increase in liver FDG uptake with elevated urine BPA levels (11). In clinical practice, visual and quantitative assessments of ^18^F-FDG PET/CT studies often utilize liver uptake values as reference or normalization factors. Various biological factors, including age, sex, weight, and serum glucose levels, can influence liver SUVs (12). Our study underscores the significance of increased BPA exposure as a crucial factor influencing liver FDG uptake.

BPS, a structural analog of BPA, is increasingly detected in human biological samples (13). Unlike BPA, our study revealed that higher BPS exposure may be associated with reduced FDG uptake in the thyroid in male. Bisphenols (BPs) are recognized as potential destructive agent of thyroid function and are commonly found in various consumer products (14). BPS exposure has been linked to disturbances in thyroid hormone synthesis pathways, potentially leading to thyroid dysfunction (15). Furthermore, research has suggested that elevated BPS levels in pregnant women may lower thyroid hormone levels, indicating a potential mechanism for reduced FDG uptake due to thyroid insufficiency induced by BPS exposure (16). Additionally, studies have associated higher BPS levels with an increased risk of thyroid cancer. These findings prompt further investigation into the potential use of FDG PET/CT imaging to predict BPS levels and assess the risk of thyroid cancer (17).

Moreover, our study observed a positive correlation in female between BPS exposure and cerebral cortex SUV_max_ and SUV_mean_. Animal experiments have demonstrated that increased BPS exposure can affect neurobehavioral responses and cognitive functions (18, 19). We hypothesize that this phenomenon may contribute to chronic inflammation of the cerebral cortex, leading to heightened FDG uptake. However, further research is needed to validate this hypothesis and elucidate the underlying mechanisms.

Certainly, our research has several limitations. Firstly, the sample size of the study is relatively small, necessitating further studies to validate the effects of BPA and BPS on major organs. It is worth noting that when the sample size is reduced after subgroup analysis, statistical efficiency may be reduced, which needs expand sample size to verify sex-dependent disruptive effect for BPA and BPS. Secondly, BPA and BPS are known to exert reproductive effects. Due to potential interference from renal urine and challenges in delineating the region of interest for bilateral ovaries, we did not analyze the relationship between ovary SUV and BPA or BPS. Additionally, it has been reported that higher concentrations of urinary BPS in women are associated with increased body fat accumulation, excluding visceral adipose tissue mass (20). However, we did not evaluate the effect of BPS on adipose tissue metabolism.

## Conclusion

Our study demonstrates that urine BPA and BPS exert differential influences on tissue metabolic parameters observed on ^18^F-FDG PET/CT imaging, particularly affecting the liver, thyroid, and cerebral cortex. This finding suggests that SUV derived from FDG uptake by these organs on ^18^F-FDG PET/CT imaging may reflect the extent of BPA and BPS exposure in the body. However, further prospective studies are needed to validate these findings.

## Data Availability

The raw data supporting the conclusions of this article will be made available by the authors, without undue reservation.
